# Clinical effect of stannous fluoride and amine fluoride containing oral hygiene products: A 4-year randomized controlled pilot study

**DOI:** 10.1038/s41598-019-44164-9

**Published:** 2019-05-22

**Authors:** C. Frese, T. Wohlrab, L. Sheng, M. Kieser, J. Krisam, D. Wolff

**Affiliations:** 1 Department of Conservative Dentistry, Clinic for Oral, Dental and Maxillofacial Diseases, Dental School, University Hospital Heidelberg, Heidelberg, Germany; 2 Institute of Medical Biometry and Informatics, Ruprecht Karls University, Heidelberg, Germany; 3 Department of Conservative Dentistry, Clinic for Oral, Dental and Maxillofacial Diseases, Dental School, University Hospital Tübingen, Tübingen, Germany

**Keywords:** Dentistry, Randomized controlled trials

## Abstract

This 4-year randomized controlled trial (RCT) aimed at investigating whether routine home use of both a SnCl_2_/AmF/NaF-containing mouth rinse and toothpaste has a preventive effect on oral health. Fifty-four test subjects were examined in biannual intervals. The primary endpoint “dental erosion” was determined by the Basic Erosive Wear Examination (BEWE). The secondary endpoints were “saliva pH”, “dentin hypersensitivity” generated by Visual Analogue Scale (VAS), and “discoloration” measured by the Lobene Stain Index (LSI). A mixed model for repeated measures (MMRM) was used to analyze the primary endpoint “dental erosion”. Primary analysis showed a significant intervention effect of the SnCl_2_/AmF/NaF-containing test product (p_1_ = 0.0242). This result was confirmed by two additional MMRM-based sensitivity analyses. Comparison of all models showed “dental erosion” values of the intervention group  below values of the control group. Discoloration of the teeth was significantly higher in the intervention than in the control group at all time points. Saliva pH and dentin hypersensitivity were not significantly different between groups over four years. In summary, this RCT is the first to indicate a possible preventive effect of SnCl_2_/AmF/NaF-containing oral hygiene products on dental erosion over a follow-up period of four years.

## Introduction

In the treatment and prevention of dental erosion, no uniform evidence-based clinical guidelines are available. Prevention measures are especially important, since there is evidence that teeth already damaged by erosion are more vulnerable to acidic attacks^1^. Studies investigating the effect of fluoride in commercial toothpastes on dental erosion confirmed the effectiveness of fluoride compared to a placebo^2,3^. However, the protective effect of fluoride on dental erosion only sets in with a higher dose of 1.25%^4,5^ and prevention of dental erosion by fluoride containing toothpaste may not be sufficiently achieved^6^. Organic substances like arginine and casein phosphopeptide-stabilised amorphous calcium phosphate (CPP-ACP) show varying results in the prevention of dental erosion^6,7^. Titanium fluoride (TiF_4_) provides promising results *in vitro*, however, long-term clinical studies that evaluate its effectiveness are still lacking^6,8^. Repeated application of stannous fluoride containing toothpaste improves protection against dental erosion^9^. The combination of tin and fluoride shows an even higher efficacy compared to the sole use of tin or fluoride^10,11^. Stannous fluoride reacts with hydroxyapatite to form CaF_2_, Sn_2_OHPO_4_, Sn_3_F_3_PO_4_, or Ca(SnF_3_)^12^. The tin ions precipitate at the tooth surface and within the acquired enamel pellicle to form a protective layer, which is more acid-resistant than pure CaF_2_^13,14^. On eroded tooth surfaces, tin is deposited in the enamel layer close to the surface^10^. As far as dentin hypersensitivity is concerned, tin-containing oral hygiene products, in addition to other formulations, also demonstrated effectiveness^15^. The clinical effect of stannous fluoride containing products was demonstrated in various *in situ* studies^10,16–18^ as well as in clinical investigations of several months duration^19,20^. For the assessment of the primary endpoint “dental erosion”, the Basic Erosive Wear Examination (BEWE) was chosen^21^. It was developed to allow monitoring and recording of the severity and progression of erosive tooth wear in general practice as well as in education and research. The secondary endpoint “dentine hypersensitivity” was recorded by the use of the 10 grade Visual Analogue Scale (VAS) on the basis of which the participants were able to record their subjective perceptions.

To the best of the authors’ knowledge, there is no randomized clinical trial with an observation period of four years in the dental literature. Therefore, according to the PICO format, the aim of this pilot study was to evaluate, whether the routine use of mouth rinsing solutions and tooth pastes that contain stannous, amine and sodium fluoride (SnCl_2_/AmF/NaF) have a positive effect on oral health with respect to dental erosion and dentin hypersensitivity in a population of athletes. In a former cross-sectional study of our research group, endurance athletes have been identified to have a higher risk of developing dental erosion than the control groups^22^. It was hypothesized that the use of both a SnCl_2_/AmF/NaF-containing mouth rinsing solution and tooth paste leads to alterations in the manifestation of dental erosion. The null hypothesis was that SnCl_2_/AmF/NaF-containing mouth rinsing solutions and tooth pastes have no effect on oral health.

## Methods

### Study design

This randomized, controlled clinical trial meets current ethical standards and was performed in accordance with the relevant directives and regulations. It was conducted after obtaining approval from the ethics committee of the Medical Faculty of the University of Heidelberg in December 2012 (S-566/2012). The study was registered at the German Clinical Trials Registry Platform (DRKS00005019, date of registration 2013/05/27) that is linked to the International Clinical Trials Registry Platform of the World Health Organization (WHO).

**Figure 1:**
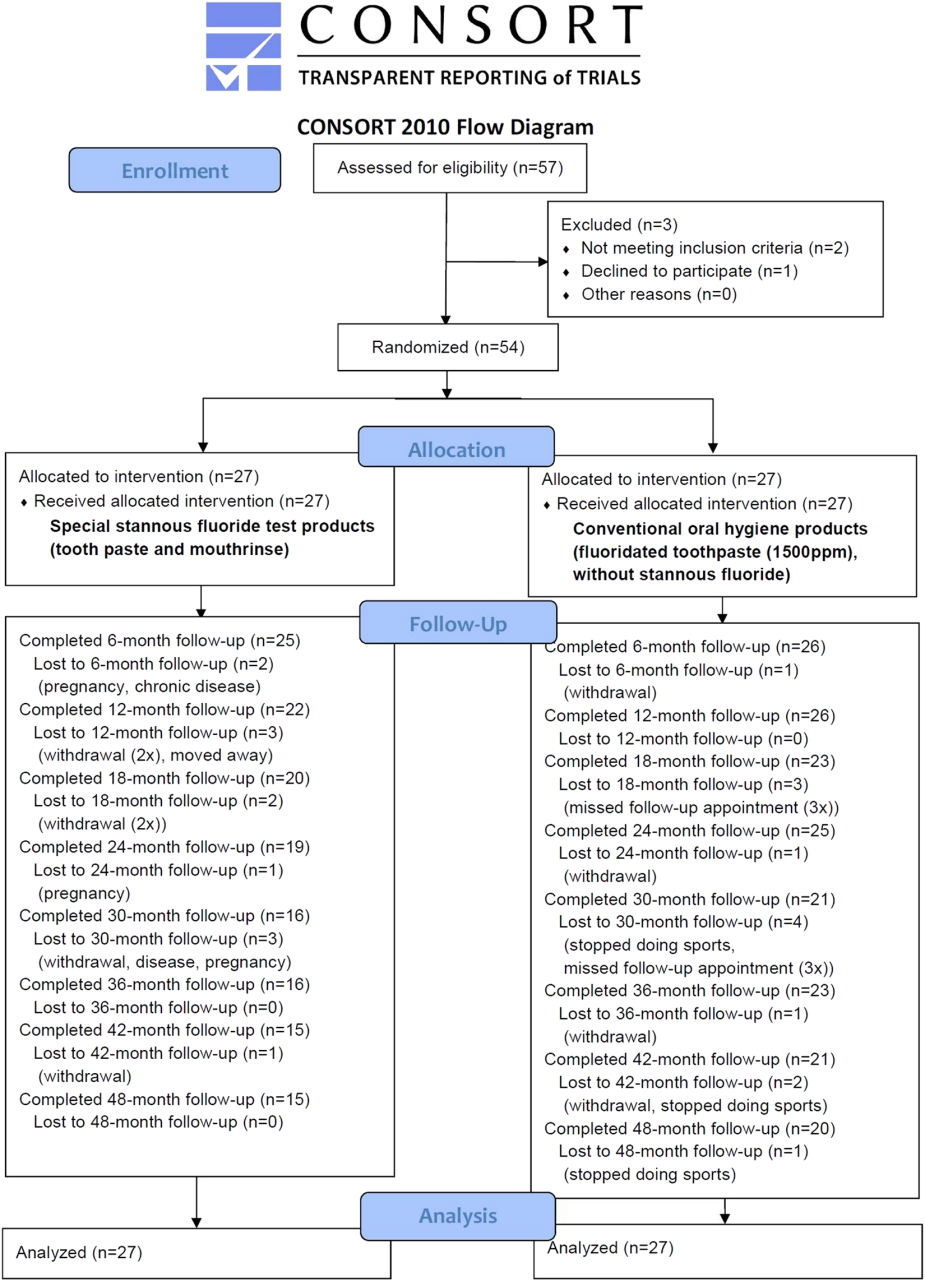
Depicts the CONSORT flow diagram, the adverse events as well as the reasons for withdrawal. With 19 patients withdrawing from the study, the overall dropout rate was 35.19%, with 7 subjects resigning from the control, and 12 subjects from the test group. Six control subjects missed one follow-up appointment each, but continued to participate in the remaining follow-up examinations afterwards^23^.

The fifty-four subjects that participated were endurance athletes from sports clubs or the university and gave written informed consent. They were randomized by block randomization (sequentially numbered envelopes) into intervention and control group (CONSORT flow diagram, Fig. 1)^23^. The random allocation sequence was generated by the statistician; enrollment and assignment of participants to interventions was done by the principal investigator. The participants in the intervention group were instructed to use a stannous fluoride-containing [(AmF)/NaF/SnCl_2_] mouth rinse (500 ppm F-, 800 ppm Sn2+), 1 × 30 s per day, a toothpaste containing NaF/Sn(2+), and the biopolymer chitosan (elmex EROSIONSSCHUTZ, CPGABA GmbH, Hamburg, Germany) for daily oral hygiene at home.

The participants in the control group did not get any products, but were instructed to use a fluoridated toothpaste (1500 ppm) along with the conventional oral hygiene products they were normally using at home. They were explicitly told not to use oral hygiene products containing stannous fluoride and the control participants provided information about the oral hygiene products they used at home at each of their follow-up appointments.

For all participants, recall examinations took place every six months. Each study appointment included medical history, completing the dental chart, assessment of dental erosion (BEWE), dentin hypersensitivity (VAS), tooth stain (LSI), as well as saliva. Study investigations were done by one blinded and calibrated examiner with binocular loupes (2.5x magnification) and an additional light source.

### Assessment of dental erosion (BEWE)

The prevalence and severity of dental erosion was assessed by the four-level Basic Erosive Wear Examination (BEWE). The most severely affected surface of each sextant was recorded, the cumulative score sum calculated and transferred to an individual risk level^21^.

### Assessment of dentin hypersensitivity (VAS)

The participants gave information on gender, height, body weight and oral hygiene regime. Frequency of brushing per day, duration of brushing in minutes and special interdental hygiene were recorded. Additionally, participants were asked whether they suffered from tooth hypersensitivity, and to quantify the degree based on a 10 grade Visual Analogue Scale (VAS).

### Assessment of tooth stain (LSI)

Tooth stain assessment was included in the study after the 12 months follow-up (starting at recall #3). The extrinsic stain on buccal and oral sites of the maxillary and mandibular anterior teeth was assessed prior to professional tooth cleaning and oral hygiene instructions^24^ using the Lobene Stain Index (LSI). Buccal and oral sites of the anterior teeth were scored for both the areas stained and the intensity of staining, according to a 0 to 3 scoring method: 0 = no stain, 1 = stain up to 1/3 of the surface area/light stain, 2 = stain up to 1/3 to 2/3 of the surface area/moderate stain, 3 = stain over more than 2/3 of the area/heavy stain.

### Saliva assessment

Saliva testing was performed using a commercial test kit (Saliva Check Buffer, GC EUROPE, Leuven, Belgium). Saliva pH was checked with paper pH strips and stimulated saliva was quantified by collecting it while chewing a piece of wax for 5 min.

### Handling of missing data

Being a RCT with a four-year observation period, this study had to deal with exclusion and withdrawal of test persons, and the subsequent loss of data. The number of subjects resigning from the study, as well as their reasons for doing so, were documented in the CONSORT flow diagram (Fig. 1)^23^. Data were analyzed according to the intention-to-treat principle (ITT), i.e. all randomized patients were included independently of protocol violations and analyzed according to the group to which they were originally assigned. Due to the Mixed Model of Repeated Measures (MMRM) approach, missing data were essentially not imputed in the primary model. However, missing data in the primary outcome were calculated by sensitivity analysis using multiple imputation.

### Statistical analysis

For the pilot study an initial sample size of n = 54 was chosen. Descriptive analyses were conducted by the information gathered from the patients and the clinical investigations of the baseline examinations. Mean, SD, median, minimum and maximum were determined for continuous variables, while absolute and relative frequencies were computed for categorical variables, along with descriptive p-values to assess the homogeneity between treatment groups (using Wilcoxon-Mann-Whitney tests for continuous variables, and chi-squared tests for categorical ones). Statistical analysis of the primary endpoint “dental erosion” was used to identify group differences. In doing so, the long-term course of the study was taken into account, as well as the resulting measurement repetitions. The primary analysis of the BEWE was performed with a linear mixed model for repeated measurements (MMRM), which was adjusted to the saliva pH value; for the BEWE at baseline an unstructured covariance matrix was used. Effect estimates and type III test-based p-values were determined along 95% confidence intervals. Sensitivity analyses included a MMRM for which the outcome data were allocated using multiple imputation by the fully conditional specification method^25^. Another MMRM used age, gender, saliva flow [ml/min], hypersensitivity by VAS, frequency of tooth brushing per day, duration of tooth brushing per day, and type of oral hygiene products as additional factors and covariates. Effect estimates for the comparison of each time points between groups were calculated by Least Squares Means statements (LSMEANS) together with the 95% confidence intervals and descriptive p-values. Boxplots and confidence intervals were plotted to illustrate the treatment effect over time. A p-value of < 0.05 was regarded as statistically significant.

The secondary endpoints saliva pH, hypersensitivity (VAS), and tooth discoloration (LSI) were analyzed analogously with linear mixed models for repeated measurements, including the treatment group, the baseline values of BEWE, VAS, LSI as factor, and covariates. The LSI was only determined after 12 months, therefore, the baseline values could not be integrated into the model. All analyses were performed by the software package SAS® System 9.4 (SAS Inc., Cary/NC, USA). RStudio Desktop 1.1.383 was used to create the graphics.

### Financial disclosure

The authors declare that there are no financial or other dependencies, which may have given rise to a possible conflict of interest at the time of conducting the study, nor having the potential to do so in future. The underlying study was financed by in-house resources. The SnCl2/AmF/NaF-containing oral hygiene products were kindly provided by the company (CPGABA GmbH, Hamburg, Germany). We acknowledge financial support by Deutsche Forschungsgemeinschaft within the funding programme Open Access Publishing, by the Baden-Württemberg Ministry of Science, Research and the Arts and by Ruprecht-Karls-Universität Heidelberg.

## Results

### Descriptive statistics of the baseline examination

The fifty-four participants at the beginning of the study were evenly distributed among the test and control group (n = 27 each). The average Basic Erosive Wear Examination (BEWE) score sum for all participants was 8.44 ± 3.11 with no difference between control and test group (p = 0.675, Table 1). Likewise, no differences were found concerning saliva pH and dentin hypersensitivity (Table 1).

**Table 1:** Descriptive display of the variables at baseline (t = 0).

Variable	Control n = 27	Intervention n = 27	Total n = 54	p-value*
**Age**				
- Mean +/− SD	36.26 +/− 8.68	34.44 +/− 10.01	35.35 +/− 9.32	0.387
- Median	36	34	35,5	
- Min, Max	22, 52	20, 57	20, 57	
**Gender**				
- Male	24 (88.9%)	17 (63.0%)	41 (75.9%)	0.026
- Female	3 (11.1%)	10 (37.0%)	13 (24.1%)	
**BEWE sextant score**				
- Mean +/− SD	8.22 +/− 3.15	8.67 +/− 3.10	8.44 +/− 3.11	0.675
- Median	8	8	8	
- Min, Max	3, 18	4, 15	3, 18	
**pH value**				
- Mean +/− SD	6.78 +/− 0.38	6.70 +/− 0.33	6.74 +/− 0.36	0.639
- Median	6.80	6.80	6.80	
- Min, Max	5.80, 7.60	5.80, 7.40	5.80, 7.60	
**Hypersensitivity measured by VAS**				
- Mean +/− SD	4.36 +/− 2.08	4.56 +/− 2.33	4.46 +/− 2.19	0.760
- Median	4	5	5	
- Min, Max	1, 9	1, 10	1, 10	

*p-values were calculated using the Wilcoxon-Mann-Whitney test for continuous variables, and the chi-squared test for categorical variables.

No difference was found between the groups where daily tooth brushing is concerned with 2.10 ± 0.42 times per day (median = 2) for a duration of 2.59 ± 0.64 minutes (median 2.5 min). 42.6% of the test subjects stated that they used dental floss, 37.0% said they did not use such tools, and 20.4% declared they used interdental brushes. All test subjects were using fluoride-containing tooth pastes, but only 29.6% additionally used fluoride-containing mouth rinsing solutions prior to intervention.

### Pattern of the missing data

Of the altogether 54 test subjects at baseline, 29 (53.70%) participated in all follow-up examinations. With 19 patients withdrawing from the study, the total dropout rate was 35.19%, with 7 subjects withdrawing from the control and 12 subjects withdrawing from the test group. Figure 1 depicts the CONSORT flow diagram, the adverse events and the reasons for withdrawal^23^. Some of the participants, being university students, moved away or were unwilling to participate any longer, two got pregnant and one participant suffered from a chronic disease and was not able to participate any longer. Six missed one follow-up appointment each, but continued to participate in the remaining follow-up examinations.

### Analysis of the primary endpoint BEWE

**Figure 2:**
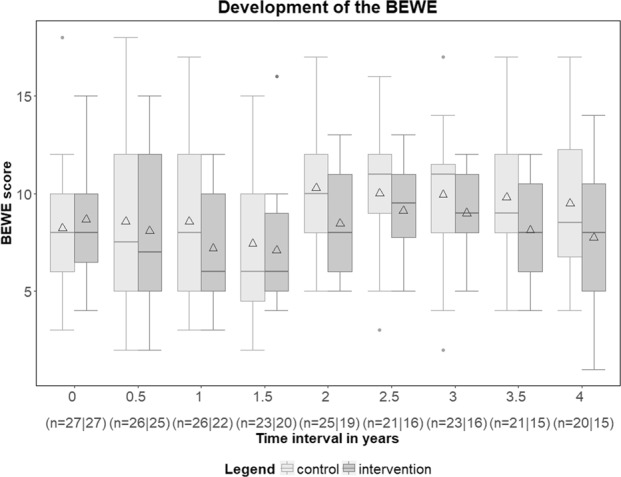
Boxplots for the primary endpoint BEWE over time. ∆ = arithmetic mean. N: Number of observations in the control group/intervention group.

**Figure 3:**
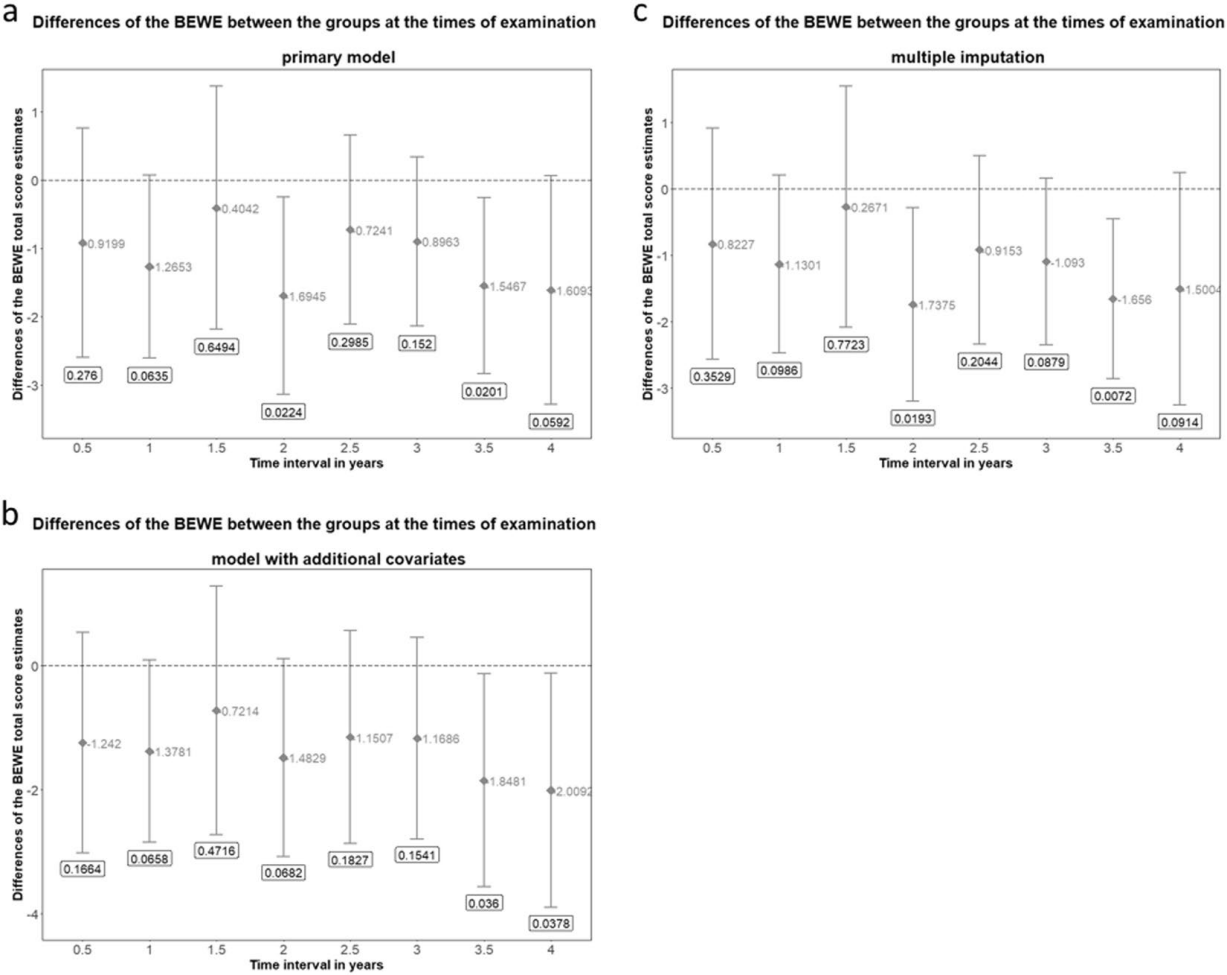
(**a**–**c**) Estimates of the least squares means with 95% confidence intervals concerning the group differences of the BEWE for all three linear models: (**a**) primary model; (**b**) model with additional covariates; and (**c**) multiple imputation. The p-value at every point in time is depicted below the respective confidence interval.

Figure 2 shows the chronological course of the BEWE from both intervention and control group. Analysis of the primary endpoint BEWE was conducted by the primary Mixed Model for Repeated Measures (MMRM) (1), the MMRM with additional covariates (2) as well as with the MMRM with multiple imputations of primary outcome data (3) (Table 2). All analyses showed significant intervention effects (p_1_ = 0.0242, p_2_ = 0.0338, p_3_ = 0.0259). A high BEWE at baseline was associated with a significantly higher BEWE at follow-up (p < 0.0001) for all three models. Of the additionally tested fixed effects in the sensitivity analysis, only gender had a significant influence on the BEWE (Table 3, p = 0.0294). Table 3 depicts the parameter estimates of the fixed effects. Figure 3a–c compares the three different statistical models for group differences at each of the examination times. Both in the primary model as well as in the model with additional covariates, the values of the intervention group were always below those of the control group. In the primary MMRM, the differences were significant at t = 2.0 (p-value = 0.0224) and t = 3.5 (p-value = 0.0201). Similar results were seen for the multiple imputation MMRM, specifically at t = 2.0 (p = 0.0193) and t = 3.5 (p = 0.0072). For the MMRM with additional covariates, differences were significant at t = 3.5 (p-value = 0.0360) and t = 4.0 (p-value = 0.0378).

**Table 2:** Results of the statistical evaluation of the primary endpoint with three models showing type 3 test-based p-values (primary and model with additional covariates as well as the model after multiple imputation).

Effect	Primary model	Model with additional covariates	Multiple imputation model
	p_1_-value	p_2_-value	p_3_-value
**Type 3 test-based p-values of fixed effects**			
Group (intervention SnCl_2_/AmF/NaF)	*0.0242	*0.0338	*0.0259
Time of examination	** < 0.0001	** < 0.0001	
Group*time of examination	0.5970	0.8113	
BEWE at t = 0	** < 0.0001	** < 0.0001	** < 0.0001
pH value	0.9650	0.7470	0.8681
Age		0.2977	
Gender		*0.0294	
Saliva flow [ml/min]		0.7937	
Hypersensitivity by VAS		0.3367

**Table 3:** Parameter estimates for fixed effects in the primary and enhanced model.

Effect	Primary model			Model with additional covariates			Multiple imputation model		
	Estimates	Standard error	p-value	Estimates	Standard error	p-value	Estimates	Standard error	p-value
**Parameter estimates for fixed effects**									
BEWE at t = 0	0.7740	0.0876	** < 0.0001	0.7563	0.1146	** < 0.0001	0.7897	0.1117	** < 0.0001
pH value	0.0115	0.2607	0.9650	−0.0906	0.2802	0.7470	0.0908	0.5464	0.8681
Age				−0.0378	0.0358	0.2977			
Gender Female				1.9508	0.8680	*0.0294			
Male				0	.	.			
Saliva flow [ml/min]				−0.0348	0.1325	0.7937			
Hypersensitivity				0.0884	0.0917	0.3367

### Analysis of the secondary endpoints

#### Saliva pH

**Figure 4:**
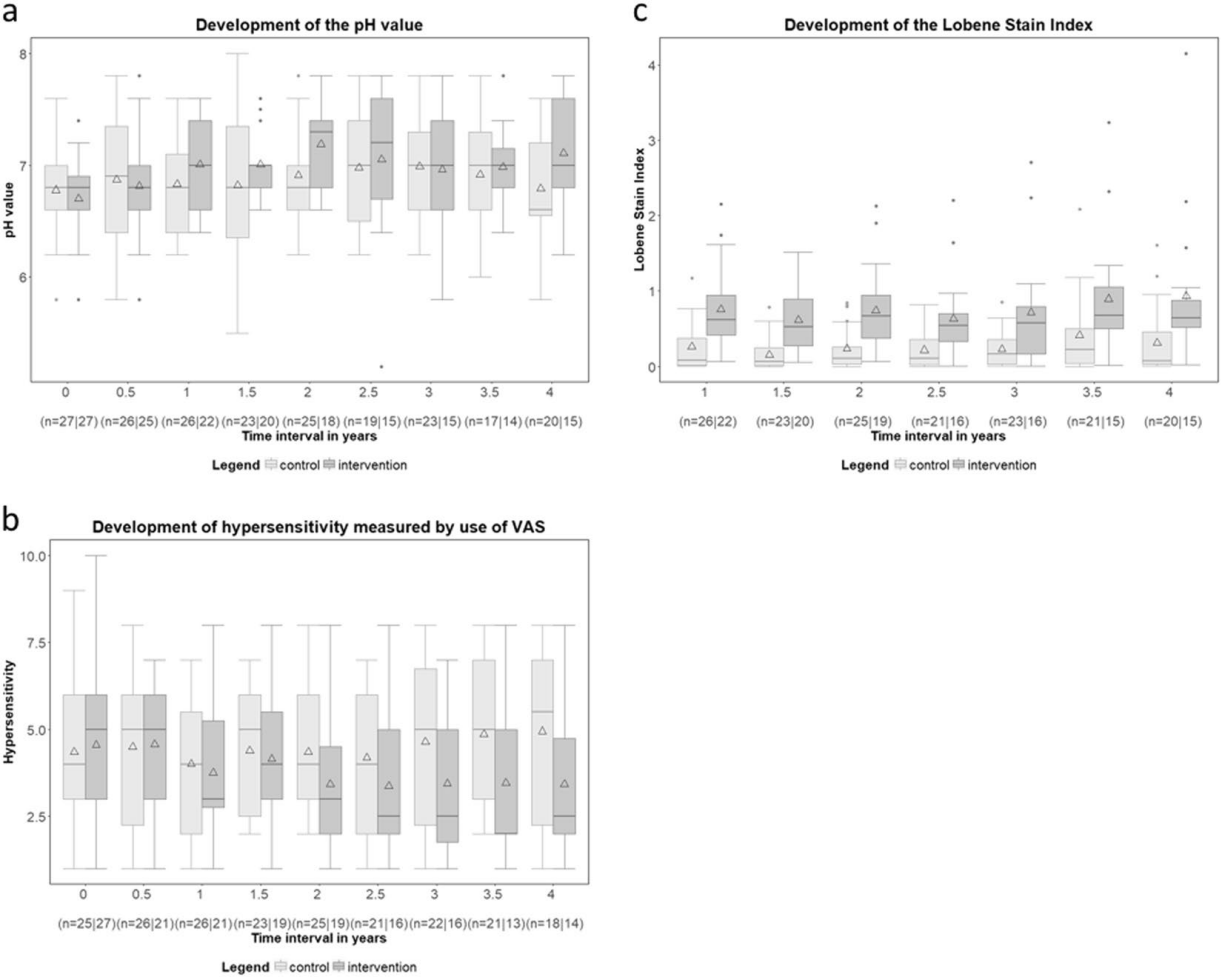
(**a**–**c**) Boxplots of the secondary endpoint saliva pH value; (**b**) Chronological sequence of the secondary endpoint hypersensitivity; and (**c**) Chronological sequence of the secondary endpoint Lobene Stain Index over time. ∆ = arithmetic mean. N: Number of observations in the control group/intervention group.

As shown in Fig. 4a, saliva pH values were subject to fluctuations over time, ranging from 5.20 to 8.00. There were no differences between the groups, nor between the investegated time points. Table 4 illustrates the sensitivity analysis, showing parameter estimates and p-values for the secondary endpoints. The saliva pH at the baseline examination was a significant predictor of the saliva pH over time (p < 0.0001). For the parameter estimates of the differences between control and intervention group, a group difference was detected at one point in time, after two years (p = 0.0267).

**Table 4:** Parameter estimates and type-III-test based p-values of fixed effects of the secondary endpoints saliva pH, dentin hypersensitivity (DHS), and discolorations (DC).

	Estimate	Saliva pH Std.error	p-value	Estimate	DHS Std.error	p-value	Estimate	DC Std.error	p-value
Group (intervention SnCl_2_)			0.1034			0.1693			*0.0010
Time of examination			0.5209			0.0970			*0.0155
Group*time of examination			0.3761			0.9323			0.7147
BEWE at baseline examination	0.0151	0.0138	0.2812	−0.0463	0.0833	0.5809	−0.0033	0.0157	0.8351
pH value at baseline examination	0.5303	0.1188	** < 0.0001	−0.1077	0.7176	0.8814	0.0860	0.1348	0.5274
Hypersensitivity at baseline examination	−0.0184	0.0180	0.3141	0.4976	0.1142	** < 0.0001	−0.0109	0.0207	0.5988

Note: Due to the variables’ cateogrical scale level, estimates for time and the treatment group*time interaction are not provided.

#### Dentin hypersensitivity

Figure 4b shows the secondary endpoint dentin hypersensitivity over time (measured by Visual Analogue Scale 0–10). As displayed in Table 4, the group difference was not significant (p = 0.1034). When considering the individual points in time by means of the least squares method, no difference was detected between groups at any time.

#### Lobene Stain Index

**Figure 5:**
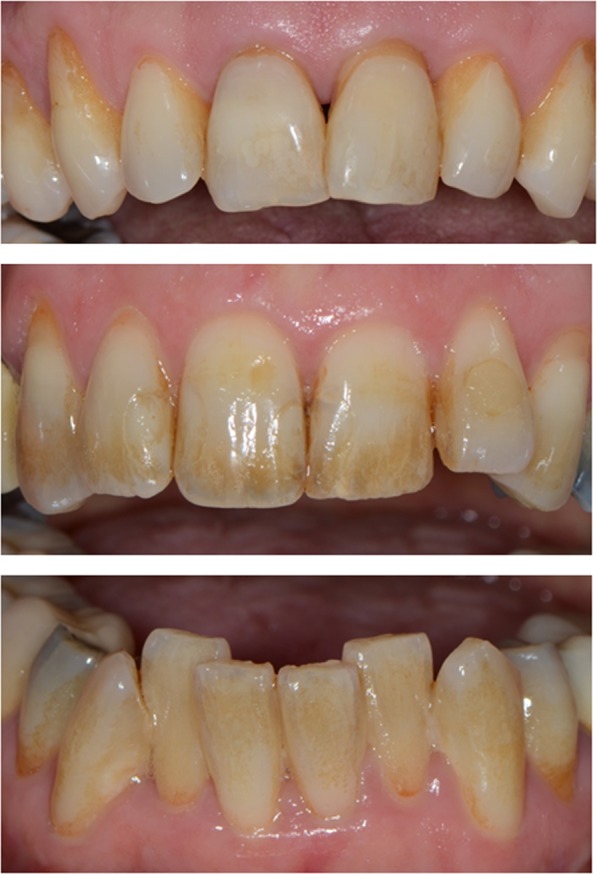
Clinical presentation of extrinsic discoloration in the test subjects, ranging from light to heavy at the examination times prior to tooth cleaning.

Figure 4c depicts the Lobene Stain Index over time showing that at any time point, the values of the intervention group were significantly higher than those of the control. As shown in Table 4, the intervention had a significant influence on the Lobene Stain Index (p = 0.0010). The values of the intervention group were estimated to be 0.4203–0.6078 higher than the values of the control group over time. Figure 5 depicts the various extents of extrinsic discolorations observed in this study.

## Discussion

This randomized controlled clinical study is the first pilot study to provide results on the clinical use of SnCl_2_/AmF/NaF-containing oral hygiene products for the prevention of oral diseases over a period of four years. The primary endpoint “dental erosion” was significantly reduced in the intervention group in all calculated models (primary MMRM: p_1_ = 0.0242; MMRM with additional covariates: p_2_ = 0.0338; multiple imputation MMRM: p_3_ = 0.0259, Table 2). Regardless of the MMRM, primary or with additional covariates, the BEWE values of the intervention group were always below those of controls (Figs 2 and 3a–c). Also, the time of investigation significantly influenced the BEWE (Table 2). Group differences in the BEWE over time showed that the preventive effect of SnCl_2_/AmF/NaF application seems to appear after a period of about 2 years (Fig. 3a–c). This is illustrated by the significant difference of the BEWE between groups after two years (primary MMRM: p = 0.0224, Fig. 3a; multiple imputation MMRM: p = 0.0193, Fig. 3c). With p = 0.0682 a tendency towards significance was seen with the enhanced MMRM at the two-year follow-up (Fig. 3b). However, significance was reached only after 3.5 years (p = 0.0360, Fig. 3b). Thus, our clinical four-year results support available *in situ* data on the preventive effect of SnCl_2_/AmF/NaF-containing oral hygiene products on dental erosion^10,17,18,26^.

Saliva pH was not influenced by the use of SnCl_2_/AmF/NaF-containing oral hygiene products. The only significant difference in saliva pH between groups was seen at t = 2.0 (p = 0.0267, Fig. 4a). The authors would like to point out the fact that there is no clinical reference data available, which would allow a discussion on this topic. These results are therefore presented as novel findings available for further debate on the relationship of clinical long-term use of SnCl_2_/AmF/NaF-containing product and saliva pH.

Dentin hypersensitivity was measured by VAS, which recorded the subjective perceptions of the patients on their sensitivities, possibly involving a distinct proportion of self-evaluative bias. Over the course of the study, the intervention group showed continuously lower values than the control group. However, type 3 test of fixed effects did not determine a significant group difference (Fig. 4b and Table 4). The control subjects were advised to continue applying their self-selected oral hygiene products. The study team merely advised that toothpastes with a fluoride content of 1500 ppm should be used and that products with stannous formulations should be omitted. This was verbally confirmed at each follow-up appointment. Whether other desensitizing substances such as products containing potassium, strontium, or arginine have been used by control subjects cannot, therefore, be completely ascertained^15^. Thus, a potentially desensitizing stannous formulation effect could have been superimposed by the effect of potentially desensitizing substances in the non-exclusively regulated variable “oral hygiene products” in the control group.

The consistently and significantly higher discolorations in the intervention group (Figs 4c and 5) showed that in the long-term clinical use, the SnCl_2_/AmF/NaF-containing oral hygiene products have the potential to react on tooth surfaces and to deposit tin-containing salts (Sn_2_OHPO_4_, Sn_3_F_3_PO_4_, and Ca(SnF_3_)_2_)^6,12^. Analogous studies on the reactive potential and preventive effect of these products were comparable^10,17,27–30^. The brown-golden discolorations, as shown in Fig. 5a–c, are typical for tin-containing products, and are caused by incorporation and interaction of the chromogens in the pellicle of the tooth. Considering the fact that the discoloration within the intervention group is steadily increasing, one could understand a progressive subject incompliance, which might have led to the higher dropout rate in this group. We have addressed this issue through repeated patient information and regular professional tooth cleaning. In spite of randomization of the subjects, there was a strong difference in the proportion of women within the groups (control n = 3 and intervention n = 10, Table 1). This fact might have led to the higher dropout, due to esthetic concerns among the higher percentage of female intervention subjects.

The weaknesses and limitations of this work arise from the number of subjects that were included due to the pilot character, and the considerable dropout rate due to withdrawal, pregnancy, and chronic illness. Also, it was challenging to ensure blinding of the examiner, because of the discolorations of teeth that occurred in the test group. To avoid this bias as best as possible, the clinical examination was conducted only after professional tooth cleaning undertaken by dental hygienist. Prior to professional tooth cleaning, this person took standardized pictures, according to which the Lobene Stain Index could be determined afterwards. Another critical issue was the assessment of dentin hypersensitivity by VAS, for it measures purely subjective characteristics. Other objective indices, for instance the Visual Erosion Dental Examination (VEDE), which would have provided more reliable information^31^, were not used in this study, due to a higher time burden. For practical reasons as well, the BEWE was only assessed by the sextant score, in order to use the simplest measuring tool for assessing dental erosion^32^.

The strength of this study lies in the inclusion of a well characterized cohort of high-risk subjects, which were randomized and longitudinally observed in a controlled design over a comparably long period of four years. The biannual follow-ups were embedded in a clinically demanding study course, requiring stringent study coordination. Adhering to the latter, detailed and valuable data on the development in each of the six months intervals could be obtained. The data demonstrated for the first time the effectiveness of SnCl_2_/AmF/NaF-containing oral hygiene products on the prevention of dental erosions in a long-term clinical setting.

## Data Availability

The authors state that with special regard to the availability of the data of this study there are no restrictions or third party interests to declare.

## References

[1] Carvalho TS, Baumann T, Lussi A (2016). Does erosion progress differently on teeth already presenting clinical signs of erosive tooth wear than on sound teeth? An *in vitro* pilot trial. BMC oral health.

[2] Barlow AP, Sufi F, Mason SC (2009). Evaluation of different fluoridated dentifrice formulations using an *in situ* erosion remineralization model. J Clin Dent.

[3] Creeth JE (2015). Dose-response effect of fluoride dentifrice on remineralisation and further demineralisation of erosive lesions: A randomised *in situ* clinical study. J Dent.

[4] Ganss C, Klimek J, Brune V, Schurmann A (2004). Effects of two fluoridation measures on erosion progression in human enamel and dentine *in situ*. Caries Res.

[5] Ganss, C., Klimek, J., Schaffer, U. & Spall, T. Effectiveness of two fluoridation measures on erosion progression in human enamel and dentine *in vitro*. *Caries Res* **35**, 325–330, 47470 (2001). 10.1159/000047470 11641567

[6] Lussi A, Carvalho TS (2015). The future of fluorides and other protective agents in erosion prevention. Caries Res.

[7] Vukosavljevic D, Custodio W, Buzalaf MA, Hara AT, Siqueira WL (2014). Acquired pellicle as a modulator for dental erosion. Arch Oral Biol.

[8] Wiegand A, Magalhaes AC, Attin T (2010). Is titanium tetrafluoride (TiF4) effective to prevent carious and erosive lesions? A review of the literature. Oral Health Prev Dent.

[9] Khambe D, Eversole SL, Mills T, Faller RV (2014). Protective effects of SnF2 - Part II. Deposition and retention on pellicle-coated enamel. Int Dent J.

[10] Schlueter N, Klimek J, Ganss C (2009). Efficacy of an experimental tin-F-containing solution in erosive tissue loss in enamel and dentine *in situ*. Caries Res.

[11] Rakhmatullina E, Beyeler B, Lussi A (2013). Inhibition of enamel erosion by stannous and fluoride containing rinsing solutions. Schweizer Monatsschrift fur Zahnmedizin.

[12] Babcock FD, King JC, Jordan TH (1978). The reaction of stannous fluoride and hydroxyapatite. J Dent Res.

[13] Ganss C, Schlueter N, Hardt M, Schattenberg P, Klimek J (2008). Effect of fluoride compounds on enamel erosion *in vitro*: a comparison of amine, sodium and stannous fluoride. Caries Res.

[14] Faller RV, Eversole SL (2014). Protective effects of SnF2 – Part III. Mechanism of barrier layer attachment. Int Dent J.

[15] Bae JH, Kim YK, Myung SK (2015). Desensitizing toothpaste versus placebo for dentin hypersensitivity: a systematic review and meta-analysis. J Clin Periodontol.

[16] Joao-Souza SH (2017). *In situ* evaluation of fluoride-, stannous- and polyphosphate-containing solutions against enamel erosion. J Dent.

[17] Ganss C, Neutard L, von Hinckeldey J, Klimek J, Schlueter N (2010). Efficacy of a tin/fluoride rinse: a randomized *in situ* trial on erosion. J Dent Res.

[18] Schlueter N, Klimek J, Ganss C (2011). Efficacy of tin-containing solutions on erosive mineral loss in enamel and dentine *in situ*. Clin Oral Investig.

[19] Nehme M (2013). A randomized clinical study investigating the staining profile of an experimental stannous fluoride dentifrice. Am J Dent.

[20] He T (2014). A clinical study to assess the effect of a stabilized stannous fluoride dentifrice on hypersensitivity relative to a marketed sodium fluoride/triclosan control. J Clin Dent.

[21] Bartlett D, Ganss C, Lussi A (2008). Basic Erosive Wear Examination (BEWE): a new scoring system for scientific and clinical needs. Clin Oral Investig.

[22] Frese C (2015). Effect of endurance training on dental erosion, caries, and saliva. Scand J Med Sci Sports.

[23] Schulz KF, Altman DG, Moher D, Group C (2010). CONSORT 2010 Statement: updated guidelines for reporting parallel group randomised trials. BMC medicine.

[24] Lobene RR (1968). Effect of dentifrices on tooth stains with controlled brushing. JADA.

[25] van Buuren S (2007). Multiple imputation of discrete and continuous data by fully conditional specification. Stat Methods Med Res.

[26] Schlueter N, Klimek J, Ganss C (2013). Randomised *in situ* study on the efficacy of a tin/chitosan toothpaste on erosive-abrasive enamel loss. Caries Res.

[27] Ganss C (2012). Efficacy of the stannous ion and a biopolymer in toothpastes on enamel erosion/abrasion. J Dent.

[28] Schlueter N (2009). Tin-containing fluoride solutions as anti-erosive agents in enamel: an *in vitro* tin-uptake, tissue-loss, and scanning electron micrograph study. Eur J Oral Sci.

[29] Schlueter N, Klimek J, Ganss C (2009). *In vitro* efficacy of experimental tin- and fluoride-containing mouth rinses as anti-erosive agents in enamel. J Dent.

[30] West NX (2012). A randomised crossover trial to compare the potential of stannous fluoride and essential oil mouth rinses to induce tooth and tongue staining. Clin Oral Investig.

[31] Mulic A (2010). Reliability of two clinical scoring systems for dental erosive wear. Caries Res.

[32] Olley RC, Wilson R, Bartlett D, Moazzez R (2013). Validation of the Basic Erosive Wear Examination. Caries Res.

